# Preoperative Planning of Spiral Intestinal Lengthening and Tailoring: A Geometrical Approach

**DOI:** 10.3390/bioengineering8020020

**Published:** 2021-01-31

**Authors:** Riccardo Coletta, Elisa Mussi, Francesca Uccheddu, Yary Volpe, Antonino Morabito

**Affiliations:** 1 Department of Pediatric Surgery, Meyer Children’s Hospital, Viale Pieraccini 24, 50141 Florence, Italy; riccardo.coletta@meyer.it (R.C.); antonino.morabito@meyer.it (A.M.); 2 Department of Industrial Engineering, University of Florence, Via Santa Marta 3, 50139 Florence, Italy; francesca.uccheddu@unifi.it (F.U.); yary.volpe@unifi.it (Y.V.)

**Keywords:** short bowel syndrome, preoperative planning, surgical simulation, pediatric, intestinal lengthening, intestinal failure

## Abstract

Short bowel syndrome is a pathological condition resulting from extensive resection of the intestine, generally performed due to congenital abnormalities, Crohn’s disease, mesenteric ischemia, or neoplasms. The main consequence of this syndrome is a reduction of intestinal absorption, which causes malnutrition and dehydration. In the most severe cases, specific and complex surgical procedures are requested to manage the syndrome. Such procedures consist of the intestinal lengthening, with lead to an increase of absorptive mucosal surface and intestinal transit time and an overall enhancement of intestinal absorption. One of the most promising surgical procedures is spiral intestinal lengthening and tailoring, which consists of a spiral incision of the intestinal wall and in the elongation longitudinally of the intestine by sliding one flap over the other. The final intestinal lengthening is strictly dependent on a series of parameters, some of which are defined by the surgeon. The present paper proposes a mathematical model, based on patient specific anatomical data, which aims to help the surgeon in defining the optimal parameters for the intervention and in foreseeing its outcomes from the preoperative planning phase. Such a tool can assist the physician in the surgery room by improving the procedure and reducing surgical times.

## 1. Introduction

Short bowel syndrome (SBS) is a severely disabling pathological condition that occurs in adults or pediatric subjects, resulting from extensive resection of the intestine, a congenital defect, infarction, or trauma. It is the most common cause of pediatric and adult intestinal failure and it is characterized by dehydration and nutrient malabsorption [1]. This disease has a considerable impact on the quality of a patient’s life [2] as well as on the national health system [3]. 

To live an acceptable life, SBS patients require intravenous or parenteral support (PS) that provides adequate fluids, electrolytes, and/or nutrients to ensure the proper functioning of the digestion, prevent permanent organ damage, avoid malnutrition, and/or dehydration-related diseases, and maintain one’s life. However, whereas the mortality of patients affected by SBS can be as high as 40% [4,5], the patient survival rate increases considerably in centers that offer structured pathways and a variety of surgical procedures tailored to the patients’ needs [6].

If an SBS patient sees no improvement from medical management, it is mandatory to consider surgical treatment to increase intestinal absorptive capacity. Surgical techniques that help these patients can be categorized under autologous gastrointestinal reconstructive (AGIR) procedures, which aim to provide better bowel physiology (in terms of remnant small bowel length and shape) and increase transit time. 

The two most common lengthening procedures are [7] the longitudinal intestinal lengthening and tailoring procedure (LILT), described by Bianchi [8], and the serial transverse enteroplasty procedure (STEP), proposed by Kim [9]. Although both procedures enable appropriate intestinal length tailoring, they suffer from limitations. LILT is considered surgically challenging because it involves the mesentery split, which can compromise the blood supply, whereas STEP modifies the muscle fiber orientation, compromising physiological intestinal motility. Furthermore, both procedures require significant bowel dilatation (40 mm and above) to be safely performed [9]. 

A new AGIR procedure, known as spiral intestinal lengthening and tailoring (SILT), was proposed by Cserni [10]. The technique does not require difficult mesenteric manipulation (whereas the LILT procedure does) and it minimally alters muscle fiber orientation compared to the STEP procedure. The ability of SILT to lengthen an intestinal segment with less bowel dilatation can open new perspectives in the surgical management of SBS patients. The technique involves a spiral incision along the intestinal tract of interest. Once the incision is made, by sliding one intestinal flap over the other, the intestine is stretched and sutured. The maximum achievable lengthening depends on some anatomical factors, on the incision angle (i.e., the helix angle, which will be described later), and on some physiological constraints. The procedure is performed by surgeons choosing standard incision angles that could be optimized with an appropriate study of the patient’s specific anatomy. To this end, the present work proposes—via the SILT procedure—a unified geometric model for the analysis, planning, and preoperative simulation of bowel lengthening surgery that allows to design, in detail, the surgery on the specific anatomy of the patient, according to the personalized medicine approach. 

## 2. Materials and Methods

Even though the intestinal tract, on which a SILT procedure is performed, is non-rigid, and not perfectly cylindrical, its schematization as a simple cylinder can help with understanding the surgical manoeuvres. During the surgery time, the exposed intestine is elongated and closed like a cylinder, and then it is re-positioned in the abdominal area according to its natural curvature. Such cylindrical schematization allows predicting and guiding the surgical cuts to obtain optimized lengthening with a known final caliber, and minimizes risks and complications. In the present study, the authors formalized the morphology of the the intestinal tract undergoing surgery with the aim of providing the surgeons with novel mathematical considerations to be used both in the preoperative planning phase than during the surgery.

### 2.1. The Geometry of the SILT Procedure

**Figure 1:**
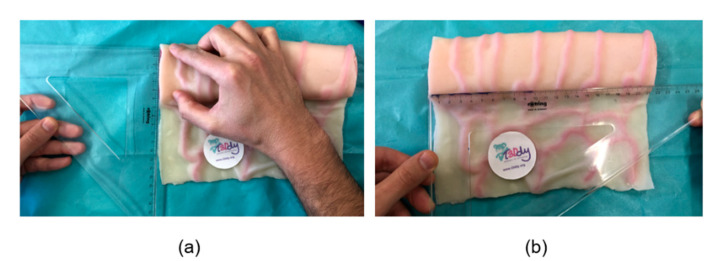
Manual measurement on the intestinal simulator of (**a**) the diameter $D_{med}$ and (**b**) length of the tract to be elongated.

To generate the three-dimensional (3D) geometrical model of the intestinal tract that undergoes the SILT procedure, such tract is hypothesized and simplified as a cylinder with a length, $L_{i}$, and a diameter, $D_{i}$, which are patient-specific parameters. Such anatomical parameters can be obtained measuring them: (1) on 3D virtual reconstruction of the patient specific anatomy (in the preoperative phase) or (2) in vivo (in the surgery room). Specifically, in the preoperative phase, the 3D reconstruction of the patient-specific bowel geometry can be obtained acquiring the anatomy with diagnostic image techniques (i.e., computed tomography scans) and isolating the region of interest with segmentation software, such as Materialise Mimics [11]. On such, 3D model measurements of $L_{i}$ and $D_{i}$ can be performed; specifically, the diameter of the intestine $D_{i}$ is defined as the average value of the two diameters measured at the extremities of the dilated intestinal tract involved in the surgical procedure. Alternatively, $L_{i}$ and $D_{i}$ can be measured in vivo directly on the patient’s anatomy in the intraoperative phase, by flattening the intestinal tube and measuring the half of its circumference (as shown in Figure 1) and the presurgical length. The measured diametric value, herein called $D_{med}$, is related to the actual cylinder diameter $D_{i}$, as in the following:

(1) $$D_{i}=\;\frac{2\cdot D_{med}}{\pi }$$

**Figure 2:**
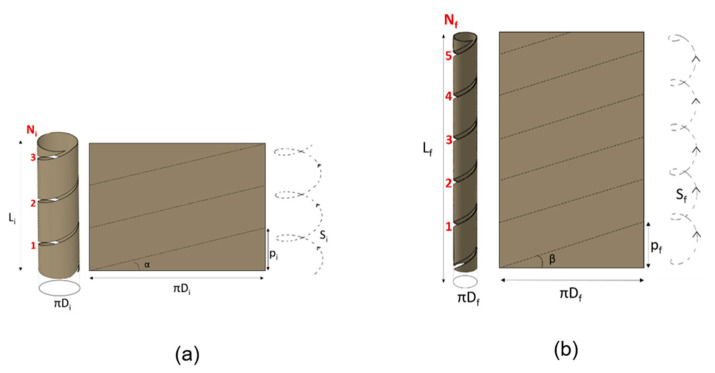
Geometrical model of the intestinal tract: (**a**) before and (**b**) after surgery.

By longitudinally cutting, the cylinder can be developed into a rectangle, where height is equal to the cylinder length ($L_{i}$) and where base is equal to the cylinder circumference $\pi D_{i}$ (see Figure 2).

Figure 2 depicts the geometrical schematization before and after surgery of the intestinal tract. The helicoidal cut is depicted in the figure, both on the cylinder and in the corresponding developed rectangle. The spiral intestinal lengthening implies, first, the choice of the helix angle α that guides the cut to unroll the tissue according to a certain helicoidal trajectory. Successively, the tube is elongated and then sutured according to a different helix angle β.

Accordingly, the known pre-surgical system variables are: (i) the patient intestinal tract diameter ($D_{i}$); (ii) its length ($L_{i}$) before surgery; and (iii) the desired diameter at the end of the procedure ($D_{f}$). As can be observed, theoretically, the intestinal surface is preserved, i.e., the surface of the cylinder before surgery corresponds to the surface of the cylinder after surgery; thus, it can be derived that the final ideal elongation $L_{f_{ideal}}\;$is known:

(2) $$\pi D_{i}\cdot L_{i}=\pi D_{f}\cdot L_{f_{ideal}}\Rightarrow L_{f_{ideal}}=\frac{D_{i}}{D_{f}}\cdot L_{i}$$

Although the theoretical elongation $L_{f_{ideal}}\;$is not influenced by the helix cutting angle α, such parameter results are relevant during the intervention since it is directly related to the number of coils to be cut $N_{i}$, and to the number of coils to be then sutured $N_{f}$. Therefore, its choice becomes critical for the success of the surgical procedure. By adjusting the number of coils to be cut ($\bar{N_{i}}$), the helicoidal cutting angle α can be derived, and a more performant surgery can be delivered in terms of surgical time. In other words, the surgeon plans and performs the surgery by optimizing the number of coils to be unrolled of the intestine wall and, afterwards, the ones to be sutured.

Practically, by leveraging the basic trigonometric formulas the number of initial spiral loops, $\bar{N_{i}}$, (before surgery) is:

(3) $$L_{i}=\bar{N_{i}}\cdot \pi D_{i}\tan\alpha \Rightarrow \;\bar{N_{i}}=\frac{L_{i}}{\tan\alpha \cdot \pi D_{i}}$$

**Figure 3:**
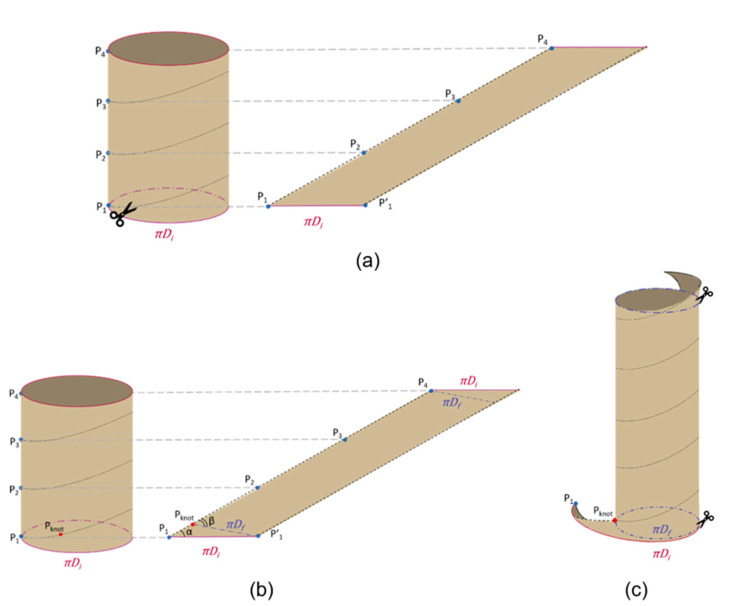
Process of the bowel lengthening: (**a**) cutting along the spiral path, (**b**) definition of the new diameter, (**c**) re-wrapping with the new diameter.

After defining the initial number of coils ($\bar{N_{i}}$), the cylinder is cut along the resulting spiral path (black dotted line in Figure 3a). Once cut, it is worth noting that, if the cylinder were completely unrolled, a parallelogram would be obtained, which base is equal to $\pi D_{i}$ (see Figure 3a). In practice, once the cylinder is cut, it is stretched and re-wrapped with the new desired diameter $(D_{f}$). In order to match the bowel flaps, physically, the parallelogram has to be re-wrapped with a new angle (β) on the base $\pi D_{f}$ (as shown in Figure 3b). In other words, if the parallelogram in Figure 3b is re-wrapped by matching $P_{1}\;$with $P_{1}^{\prime }$ the original cylinder is obtained, while if the parallelogram is re-wrapped by matching $P_{knot}$ with $P_{1}^{\prime }$ it results in the final cylinder. In this case, it is worth it to note that, at the extremities, two triangles will result in excess (as shown in Figure 3c). 

**Figure 4:**
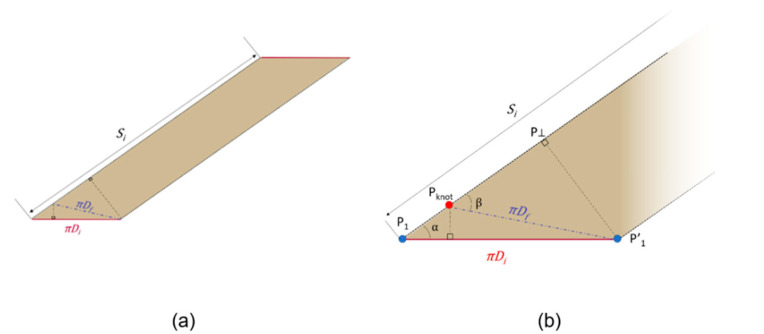
Evaluation of initial and final cylinder: (**a**) unrolled cylinder and (**b**) magnification of the base.

The trigonometric relationships among the triangles $\Delta \;P_{1}P_{1}^{\prime }P_{knot}$ and $\Delta \;P_{knot}\;P_{1}^{\prime }\;\;P_{⊥}$ (see Figure 4) allow the calculation of the angle β as reported in the following formulas:

(4) $$\{\begin{array}{c} \bar{P_{⊥\;}P_{1}^{\prime }}=\pi D_{i}\cdot \sin\alpha \\ \bar{P_{⊥\;}P_{1}^{\prime }}=\pi D_{f}\cdot \sin\beta \end{array}\Rightarrow \sin\beta =\sin\alpha \;\frac{D_{i}}{D_{f}}$$

and the calculation of the length $\bar{P_{1}P_{knot}}$:

(5) $$\{\begin{array}{c} \bar{P_{1}P_{⊥}}=\pi D_{i}\cdot \cos\alpha \\ \bar{P_{knot}P_{⊥}}=\pi D_{f}\cdot \cos\beta \end{array}\Rightarrow \;\bar{P_{1}P_{knot}}=(\pi D_{i}\cdot \cos\alpha -\pi D_{f}\cdot \cos\beta )$$

The definition of the length $\bar{P_{1}P_{knot}}$ is crucial to identify the point $P_{knot}$, key point to re-wrap the intestine during surgery, as explained in the Results section. 

**Figure 5:**
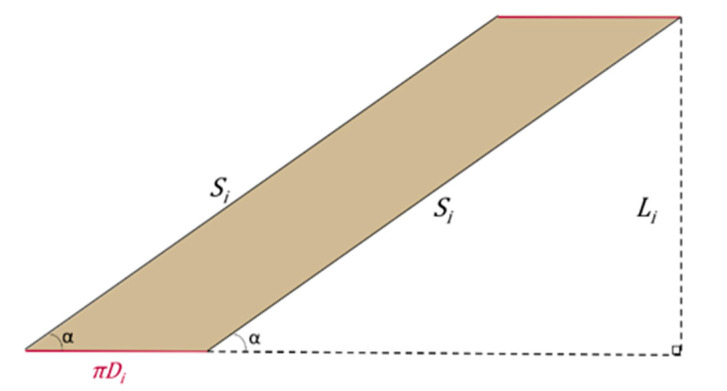
Geometrical relationships between cutting angle α, spiral path resulting and, initial length of the cylinder.

Figure 5 shows the unrolled cylinder, highlighting some geometrical relationships useful to calculate the elongation obtainable after re-wrapping.

The spiral length $S_{i}$ is related to $L_{i}$ and α according to the following equation:

(6) $$S_{i}=\frac{L_{i}}{\sin\alpha }$$

However, taking into account the triangle that must be removed for a correct re-wrapping of the cylinder with the new diameter (as shown above in Figure 4b), the total final spiral length, defined as $\hat{S_{f}},\;$results as:

(7) $$\hat{S_{f}}=S_{i}-\bar{P_{1}P_{knot}}$$

Therefore, the achievable intestine length, according to Equations (2) and (7) will be:

(8) $$\hat{S_{f}}=S_{i}-\bar{P_{1}P_{knot}}$$

Similar to Equation (3), the resulting final loops $N_{f}$ is equal to:

(9) $$N_{f}=\frac{\hat{L_{f}}}{tan\beta \cdot \pi D_{f}}$$

### 2.2. Surgical Feasibility

The surgical feasibility of the SILT technique imposes other geometric limits that must be respected and taken into account during the preoperative planning and during the surgery. In other words, the aim of the surgery is to lengthen the intestinal tract affected by the surgery as much as possible; however, previous studies, and some physical considerations, lead to the definition of some constraints. In fact, Cserni et al [10] tested the technique on animal models, which showed that it is not possible to reduce the intestinal lumen below a certain threshold (<20 mm) as this can contribute to bowel obstruction. Moreover, the cutting angle cannot be too acute; in fact, this would mean the handling of a large number of coils, which could make the surgery more complex and longer. In addition, the SILT procedure provides that, at the points where the spiral meets the mesentery, an incision in the mesentery be made. These incisions are parallel to the vessels, not compromising blood supply to the intestine, and are essential to allow the mesentery to roll over the SILT segment, allowing the intestinal wall to slide along the spiral path. However, a high number of incisions (due to a high number of coils) could lead to the cutting of the vessels of the mesentery and determine the collapse of the intestine. For these reasons, it is necessary that the cutting angle is not too acute, resulting in a high number of coils. Finally, the spiral cut could start on the mesenteric or antimesenteric flap. However, from a physical point of view, it is useful that the flap on which $P_{knot}$ is placed and which then moves on $P_{1}$ is antimesenteric because it has greater mobility not being directly connected to the mesentery. Hence, the cut must begin on the mesenteric side.

## 3. Results

**Figure 6:**
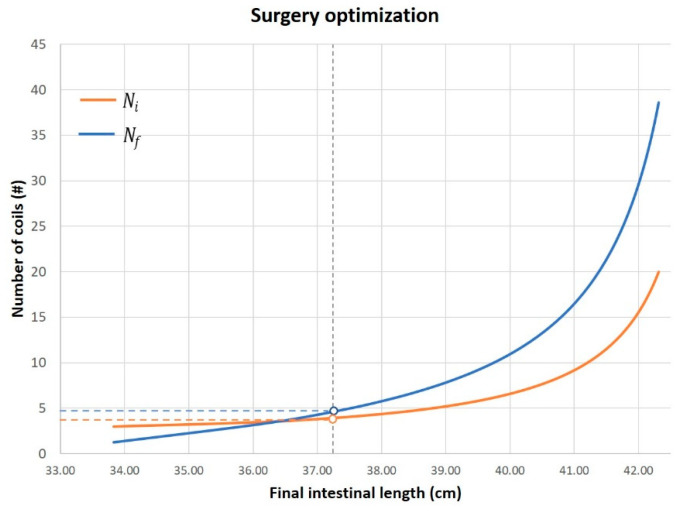
Trend of the theoretical final length and number of coils ranging the α cutting angle.

With such a mathematical framework at hand, and taking into account the described considerations on surgical feasibility, it is possible to set up the cutting angle α, to achieve the longest final length, by guarantying the desired intestinal diameter and a manageable number of coils to be cut and sutured. In fact, starting from the mathematical framework, a graph that supports the surgeon in the choice of the cutting angle can be create for each clinical case. The graph is patient-specific as it is based on the patient’s anatomical data (see Figure 6).

The graph plots the trends of the initial and final coils as a function of the final length, after fixing the values of initial length, initial diameter, and final diameter of the patient’s bowel. The graph shows a diverging behavior of the curve; thus, suggesting to keep the achievable length slightly lower than the theoretical one to significantly save surgical time and complexity. Therefore, the surgeon, based on surgical feasibility, can decide $N_{i}$ and, thus, predict the final achievable length. 

Surgical outcome, thus, arises from a trade-off between the maximum lengthening of the intestine and the number of coils. The outcome of the final intestine can be predicted in the preoperative phase through a combination of 3D anatomical reconstruction from diagnostic images and the mathematical theorization of the anatomy, as described in this work.

Specifically, preoperative planning is a key step of this surgery as, in this phase, the surgical team analyzes the clinical case, taking into account all anatomical aspects and surgical feasibility, studying the patient-specific surgical treatment. 

**Figure 7:**
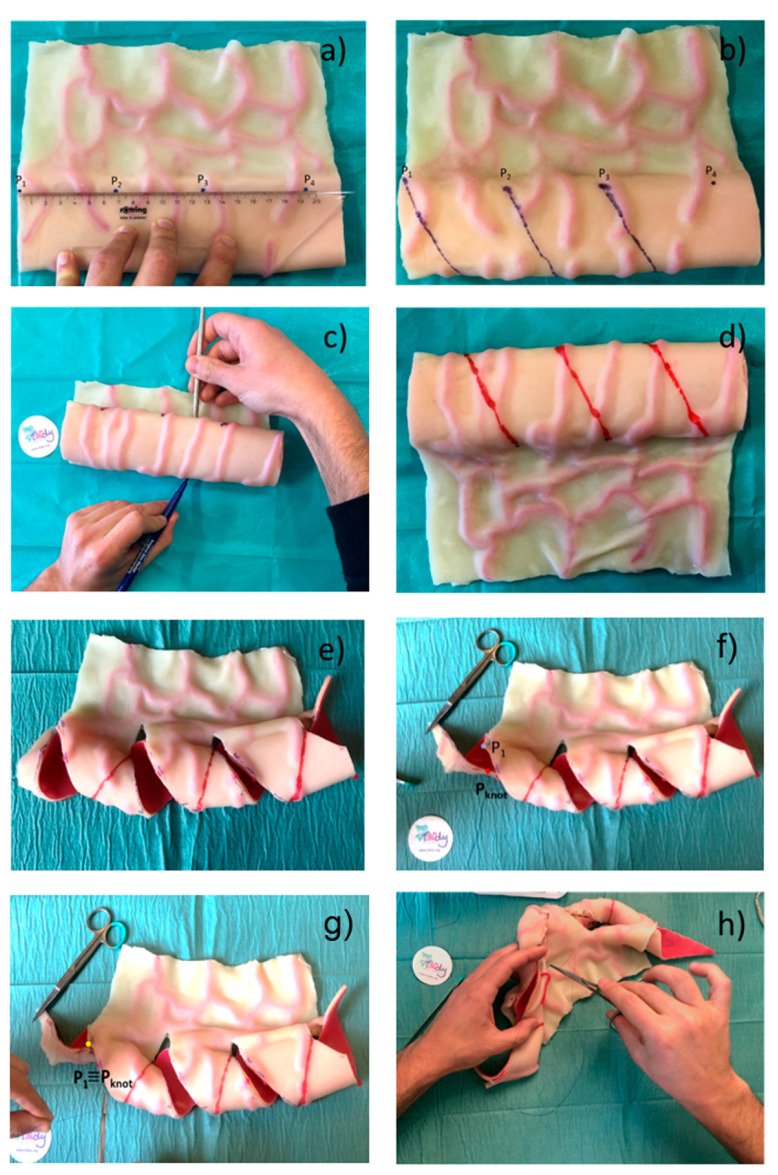
Surgical procedure. (**a**) Tracing of the points that define the helical path and (**b**) the helical path, (**c**) bowel turning up, (**d**) tracing of the helical path on the posterior wall, (**e**) result of the bowel cutting along the traced spiral path, (**f**) tracing of the $P_{kont}$, (**g**) placing of the first knot, (**h**) suturing.

To obtain a precise cut and to execute it in a simple way, the surgeon traces the spiral path on the intestinal tract to be treated with a surgical skin marker. Specifically, starting from the extremity of the intestinal tract, points $P_{1}$,…,$\;P_{n}$ and the helicoidal path are marked, considering that the segment $\bar{P_{1}P_{2}}$ corresponds to the pitch of the helix i.e. $p_{i}=\frac{L_{i}}{N_{i}}$ (see Figure 7a,b). Then, the bowel is turned upside down to access the posterior wall, and the helical path, traced in the previous phase, is continued (Figure 7c,d). The bowel is then cut along the spiral path and perpendicular incisions are made on the mesentery to allow the subsequent stretching. Another point $P_{knot\;}$is marked along the spiral path on the antimesenteric flap is at a specific distance from point $P_{1}$ (see Equation (5)); this segment defines the intestinal triangle (see Figure 4b), which must be invaginated or removed. Point $P_{knot}$ is moved until it matches the point $P_{1}$ on the mesenteric flap and the first knot is placed (Figure 4g), which allows the intestine to roll up with the correct angle β. Finally, the flaps of the intestine are sutured following the rolling angle that comes out of the first suture point.

The proposed mathematical framework plays a fundamental role in the simplification of the surgical procedure since it guides the surgeon in rewrapping the bowel after the cut, providing the exact point at which the first suture point ($P_{knot}$) must be placed to achieve the planned surgical result.

To test the proposed mathematical framework, a surgical procedure was simulated on a bowel physical replica. The simulator was manufactured by T3Ddy Lab and it reproduces a bowel tract, where length ($L_{i}$) is equal to 270.00 mm and diameter ($D_{i}$) is equal to 65 mm. Fixing the value of the final diameter to 32.5 mm, the graph (as in Figure 6) was created. The surgeon chose, based on the graph, to cut along a spiral path with an $N_{i}$ equal to four coils, predicting a final bowel length equal to 36.4 mm. The simulation of the surgery led to an intestinal tract length equal to 37.3 mm; thus, resulting in a 2% error with respect to the predicted value. This can be explained by the fact that our model does not take into account the elasticity of tissue that may have been stretched during the surgical simulation.

## 4. Discussion and Conclusions

The present work proposes a planning tool for bowel lengthening surgery performed using the SILT surgical technique in subjects affected by SBS. The surgery, first described in [12], involves the spiral incision of the intestinal wall at an angle of 45° to 60°, the longitudinal stretching, and rotation of the two intestinal flaps until they meet, and the suturing of them. The surgery is nowadays performed manually by surgeons without any instrument that could assist in the selection of the optimal incision angle of the spiral that defines the achievable final bowel length. In this study, a tool is presented which, taking into account the patient’s specific anatomy, has the ambition to assist the surgeon during the preoperative and intraoperative phase in selecting the best angle incision, allowing the optimization of intestinal lengthening and predicting the final lengthening achievable.

The developed tool, based on a simplification of the patient-specific intestinal anatomy, and a mathematical model describing the surgical procedure, allows for simplification of the surgical procedure by giving the physician accurate measurements to assist him during the surgical procedure.

In the previous reported publications on SILT, either an intestinal double layer simulator or animal model were used, but in both these studies, no clear indication about where to cut the bowel spirally was described [12,13]. The main limits of the previous publications on the SILT technique regards the lack of a clear description on how the surgeon decides where to cut the bowel in order to reshape it in a spiral fashion. This lack of clarity can reduce the expansion in the use of this novel surgical procedure. This study tries to sort out the limitation behind the SILT procedure by creating and validating a mathematical model that can be easily used. Using the indication proposed by the tool, taking into account the patient specific anatomy, the surgeon can easily pick how much of the bowel segment needs to be lengthened, but most importantly, the surgeon can improve the surgery results by optimizing the incision and obtaining greater precision. Among the strengths of this study is that it allows the simplification of a particularly complex procedure, making it accessible to a wider range of surgeons and, thus, enables a broader spectrum of patients to benefit from such a treatment. Future studies are envisaged 1) to validate the proposed tool by testing the surgical procedure on animal models, and 2) to improve the simplified model of the intestinal anatomy, that, to date, does not take into account, for example, potential changes in the diameter along the intestinal tract involved in the surgery.
